# Diagnostic value of anti-cyclic citrullinated peptides and association with HLA-DRB1 shared epitope alleles in African rheumatoid arthritis patients

**DOI:** 10.1186/ar2945

**Published:** 2010-03-02

**Authors:** Madeleine Singwe-Ngandeu, Axel Finckh, Sylvette Bas, Jean-Marie Tiercy, Cem Gabay

**Affiliations:** 1 Unit of Rheumatology, Department of Internal Medicine, School of Medicine, University of Yaoundé, Yaoundé, Cameroon; 2 Division of Rheumatology, University Hospitals of Geneva, 26 Avenue Beau-Séjour, 1211 Geneva 14, Switzerland; 3 National Reference Laboratory for Histocompatibility, Division of Immunology and Allergy, University Hospitals of Geneva, 4 rue Gabrielle Perret-Gentil, 1211 Geneva 14, Switzerland; 4 Department of Genetics and Laboratory Medicine, University Hospitals of Geneva, 4 rue Gabrielle Perret-Gentil, 1211 Geneva 14, Switzerland; 5 Department of Pathology & Immunology, University of Geneva School of Medicine, 1 rue Michel-Servet, 1211 Geneva 4, Switzerland

## Abstract

**Introduction:**

The purpose of this study was to examine the diagnostic performance of autoantibodies against citrullinated peptides/proteins (ACPA) and to determine the prevalence of HLA-DRB1 shared epitope alleles (SE) in African patients with rheumatoid arthritis (RA).

**Methods:**

Serum levels of anti-cyclic citrullinated peptides antibodies (anti-CCP2, anti-CCP3), IgM and IgA rheumatoid factors (RF) were measured by enzyme-linked immunosorbent assay in the serum of 56 consecutive RA patients regularly followed in the Rheumatology Unit of the School of Medicine, University of Yaoundé, Yaoundé, Cameroon. Genotyping of HLA-DRB1 alleles was performed by polymerase chain reaction and hybridization with sequence-specific oligonucleotide probes on microbeads arrays. Fifty-one patients with other inflammatory rheumatic diseases and 50 healthy individuals were included as controls.

**Results:**

An anti-CCP2 assay showed the best diagnosis sensitivity (82%) and specificity (98%) with high positive predictive (PPV) (96%) and negative predictive values (NPV) (91%). Thirty percent of RA patients were carrying at least one copy of the HLA-DRB1 shared epitope (SE) compared to 10% and 14% of patients with other inflammatory rheumatic diseases and healthy individuals, respectively. The presence of the SE was associated with the production of ACPA.

**Conclusions:**

Anti-CCP2 antibodies are useful markers of RA in African patients. In this cohort, the prevalence of the SE is higher in RA patients than in controls but lower than that reported in patient cohorts of European ancestry. The discrepancy between the high prevalence of ACPA-positive patients and the relatively low number of SE-positive cases suggest that, in addition to SE, other genetic factors control the development of ACPA in African RA patients.

## Introduction

Rheumatoid arthritis (RA) is characterized by inflammation of the synovial membrane of diarthrodial joints leading to tissue destruction and severe disability. The cause of RA is unknown but genetic susceptibility and environmental factors appear to be involved. RA is the most frequent systemic autoimmune inflammatory disease with a prevalence of approximately 0.5 to 1% in populations of European ancestry. However, it appears to have a relatively lower prevalence among African populations, particularly those living in rural settings [1-3].

Two important autoantibody systems have been described in RA, including rheumatoid factors (RF) directed to the Fc fragment of IgG and autoantibodies against citrullinated peptides/proteins (ACPA). RFs are well-known autoantibodies associated with RA and are present in approximately 70 to 80% of RA patients, but because they are also detected in patients with other autoimmune diseases as well as in chronic infections and in lymphoma or other tumoral processes, they have limited specificity. ACPA such as anti-cyclic citrullinated peptides (anti-CCP) are directed to antigens that contain arginyl converted to citrullyl residues by peptidylarginyl deiminase enzymes [4,5]. Several studies have shown that these antibodies are present in 60% to 80% of Caucasian RA patients with a high specificity of more than 95% [6]. However, there are no data regarding the presence of these antibodies in African patients with RA.

The genetic component of RA has been determined with heritability estimates of 50% to 60% [7]. The major susceptibility loci associated with susceptibility to RA were identified approximately 30 years ago and consist of the human leukocyte antigen (HLA) class II molecules. There is extensive evidence that some *HLA-DRB1 *alleles, including HLA-DRB1*0101, HLA-DRB1*0102, HLA-DRB1*0401, HLA-DRB1*0404, HLA-DRB1*0405, HLA-DRB1*0408, HLA-DRB1*0410, HLA-DRB1*1001, HLA-DRB1*1402 are associated with susceptibility to RA. These alleles share a common amino acid sequence (QKRAA, QRRAA, or RRRAA), also termed *shared epitope *(SE), located at positions 70 to 74 within the third hypervariable region of *DRB1*, forming part of the antigen-binding site. The shared epitope accounts for at least 30% of the total genetic susceptibility [8]. In addition, the associations between the SE and other genetic markers including *PTPN22*, *CTLA4*, *CD40 *genes, the *TRAF1/C5 *region and SNPs between *OLIG3 *and *TNFAIP3 *genes, and anti-CCP positivity have been reported in different populations (reviewed in [9]).

The objective of this study was to examine the prevalence of ACPA detected by anti-CCP2 and anti-CCP3 enzyme-linked immunosorbent assays (ELISAs), and that of HLA-DRB1 alleles in African RA patients in order to examine first the diagnostic performance of these serological tests as compared to RF, and then the distribution of the SE alleles and their association with ACPA.

## Materials and methods

### Patients

This study was carried out on 56 RA patients recruited consecutively from the outpatient Rheumatology Clinic of Yaoundé Central Hospital in Cameroon. These RA patients fulfilled the American College of Rheumatology 1987 criteria for RA [10]. Fifty-one patients (20 females) with other rheumatic conditions and ages ranging from 16 to 65 (median 28), and 50 healthy individuals (33 females) with ages ranging from 22 to 55 (median 34) were included as controls. Patients with other inflammatory rheumatic conditions were consecutively recruited from the same outpatient clinic, while healthy controls were recruited among medical students and hospital workers in Yaoundé. Patients with RA were treated with disease modifying antirheumatic drugs (DMARDs), including methotrexate, hydroxychloroquine, sulphasalazine, leflunomide, combinations of methotrexate, hydroxychloroqine and sulphasalazine and/or oral prednisone. RA patients were assessed for demographic characteristics, disease duration, duration of morning stiffness, pain by visual analogue scale, number of tender joints, number of swollen joints, the presence or absence of nodules, extra-articular manifestations, and co-morbidities. The disease activity score (DAS28) was calculated as previously described [11]. Hand radiographs were obtained for each RA patient. Approval of the Cameroon National Ethical Committee was obtained prior to the study and an informed consent was obtained from all patients and controls included in this study.

### IgM and IgA RF determinations by enzyme immunoassays

Commercially available ELISA kits, purchased from Inova Diagnostics (Ruwag, Zurich, Switzerland), were used to detect IgM and IgA RF. The assays and calculations were performed according to the manufacturer's instruction. Each kit included its own RF standard and the results were calculated as arbitrary units/ml. The diagnostic performance of these kits has been previously evaluated in a Swiss population of RA patients and controls [12].

### Anti-cyclic Citrullinated Peptide antibody determination by enzyme immunoassay

The ELISA kits detecting the IgG anti-CCP2 antibodies (Immunoscan RA: regular, second generation of CCP antigen), were purchased from Euro-Diagnostica (Pharma Consulting, Burgdorf, Switzerland), and those detecting the IgG anti-CCP3 (Quanta Lite CCP3: third generation of CCP antigen), were purchased from Inova Diagnostics (Ruwag, Zurich, Switzerland). The assays and calculations were performed according to the manufacturers' protocols. Each manufacturer uses its own anti-CCP calibrator and the results were calculated as arbitrary units/ml. In addition, to avoid false positive results for anti-CCP2 antibody determination, reactivity to non-citrullinated peptides containing arginyl instead of citrullyl residues was also tested. Diagnosis sensitivity and specificity of anti-CCP2 antibody determination have been previously determined in our laboratory in Swiss and in French patients with RA and in controls [13,14]. The sensitivity and specificity of anti-CCP3 were compared to those of anti-CCP2 in two studies on different populations [15,16].

### HLA-DRB1 genotyping

Genomic DNA was extracted from 350 μl-aliquots of frozen blood samples by using the GenoM6 magnetic bead-based workstation. HLA-DRB1 generic typing was performed by PCR-SSOP (sequence-specific oligonucleotide probes) reverse hybridization using the Luminex technology after locus-specific exon 2 amplification. The method is based on fluorescent microbeads coated with oligonucleotide probes specific for the polymorphic positions of DRB1 exon 2 sequences (LabType RSSO2B HD, OneLambda). Automated reading (Labscan TM100, Luminex, Austin, Texas USA)) and interpretation led to HLA-DRB1 high resolution (four-digit) typing as previously described [17].

### Statistical Analysis

The disease characteristics of RA patients (Table 1) were described using standard non-parametric statistics (median and interquartile ranges) for continuous outcomes and percentages for dichotomous outcomes.

**Table 1:** Baseline characteristics of RA patients

Age (yrs)	53.5 (39 to 61.5)
Female sex (%)	95
Disease duration (yrs)	3 (2 to 6)
Erosions (%)	44
Subcutanous nodules (%)	7
DAS28	4.72 (3.8 to 6.4)
Morning stiffness (minutes)	30 (10 to 60)
VAS-pain (0 to10)	5 (3 to 7)
CRP (mg/L)	12 (6 to 35)
Tobacco, N (%)	1 (2)
Prednisone, N (%)	51 (91)
Dose (mg/day)	10
Methotrexate, N (%)	43 (77)
Dose (mg/day)	10
Sulfasalazine, N (%)	7 (12)
Azathioprine, N (%)	2 (5)
Leflunomide, N (%)	2 (5)
D-penicillamine, N (%)	1 (2)

Continuous values are presented as median (interquartile range 25 to 75); CRP, C-reactive protein; DAS, disease activity score; VAS, visual analogue score

The diagnostic performances and cut offs of all the serological assays used in this study have been initially validated in Caucasians. Thus, we decided to identify the most discriminant cut-off values of the different tests for this particular population, which were operationally defined as the cut-off values leading to the highest percentage of correctly classified patients. The most discriminant cut-offs to be considered as positive were with values ≥ 22 units/ml for IgM RF, ≥ 1 unit/ml for IgA RF, ≥ 32 units/ml for anti-CCP2, ≥ 17 units/ml for anti-CCP3, respectively. Using these established cut-offs, we computed the sensitivity, the specificity, the positive predictive value (PPV) and the negative predictive value (NPV) of the various biologic tests in this population. We used the area under the curve (AUC) of the receiver operating curves (ROC) to compare the diagnostic performance of the various biologic tests in this population. Sensitivity and specificity were compared using the exact McNemar's probability test.

Finally, we examined the agreement or correlation between these biomarkers using a kappa statistic. We then examined which of these tests provided independent information for the diagnosis of RA. We first performed simple stratified analyses (patients stratified according to their genetic SE status) and then performed a multivariate logistic regression model, where the diagnosis of RA was the dependent variable and the various tests the independent variables. All statistical tests were two-sided and at the 0.05 significance level. The statistical analysis was performed with STATA v. 9.1.

## Results

This study included all the RA patients followed at the University Hospital outpatient clinic of Yaoundé, Cameroon. The clinical characteristics of these 56 patients are described in Table 1. Most of them were female with established disease (more than two years). All of them had either moderate or active disease according to DAS28 levels and 44% had radiographic signs of joint erosions on hand X-rays. Ninety-one percent of patients were on DMARDs and the vast majority of them were on methotrexate (77%). Seven out of 56 patients received a combination of DMARDs but none of them were on biologic therapy as these drugs are not available in Cameroon. Fifty-one control patients with inflammatory rheumatic diseases were recruited from the same outpatient clinic and had disease duration ranging from 1 to 10 years (median 3). The different diagnosis included 18 unclassified oligoarthritis (some cases with probable reactive arthritis), thirteen patients with ankylosing spondylitis, three psoriatic arthritis, nine systemic lupus erythematosus, three oligoarthritis in HIV-positive patients, three adult Still's disease, one systemic sclerosis, and one adult with juvenile idiopathic arthritis.

The serological characteristics of the three tested groups and the sensitivity, specificity, PPV and NPV, and the AUC of the ROC of the different serological tests for the diagnosis of RA are described in Table 2. IgM RF and IgA RF were detected in 77% and 84% of RA patients with a specificity of 93% and 92%, respectively. Anti-CCP2 antibodies were present in 82% of RA patients and detected in only one subject (2%) from either of the control groups. Thus, anti-CCP2 antibodies had a high NPV and PPV and a high diagnostic performance as assessed by the AUC of the ROC (0.91, 95% CI 0.85 to 0.96). Of note, we did not detect any reactivity to control non-citrullinated peptides containing arginyl instead of citrullyl residues. Anti-CCP3 antibodies were less sensitive and specific than anti-CCP2 antibodies, but the difference was not statistically significant.

**Table 2:** Serological and immunogenetic characteristics in RA patients, controls with inflammatory rheumatic diseases, and healthy individuals

Laboratory values	RA	IRD (n = 56)	HI (n = 51)	Sensitivity (n = 50)	Specificity	PPV	NPV	AUC (ROC) (95%) CI
RF IgM (%)	43	3 (6)	4 (8)	77	93	86	88	0.85 (0.79 to 0.91)
RF IgA (%)	47	8 (16)	0 (0)	84	92	85	91	0.88 (0.82 to 0.94)
Anti-CCP2 (%)	46	1 (2)	1 (2)	82	98	96	91	0.90 (0.85 to 0.96)
Anti-CCP3 (%)	43	4 (8)	1 (2)	77	95	90	88	0.86 (0.80 to 0.92)
SE 1 or 2 copies (%)	17	7 (14)	5 (10)	30	88	59	70	0.59 (0.52 to 0.66)
- SE 1 copy (%)	15	7 (14)	5 (10)	27	88	56	68	0.57 (0.51 to 0.64)
- SE 2 copies (%)	2	0 (0)	0 (0)	4	100	100	65	0.52 (0.49 to 0.54)

Anti-CCP, anti-cyclic citrullinated peptides; AUC, area under the curve in the ROC analysis; HI, healthy individuals; IRD, inflammatory rheumatic diseases; RA, rheumatoid arthritis; RF, rheumatoid factor; Sensitivity, the percentage of RA patients who would be identified as having RA by the laboratory tests (*positive test results*); Specificity, the percentage of control patients (IRD and HI together) who would be identified as not having RA by the laboratory tests (*negative test results*); PPV, positive predictive value or the proportion of RA patients with *positive test results *who are correctly diagnosed as having RA; NPV, negative predictive value or the proportion of control patients with *negative test results *who are correctly diagnosed as not having RA; SE, shared epitope

Of the 157 DRB1-typed samples a total of 21 alleles were identified. The following two allele groups were not resolved because the differences are located in the third exon: DRB1*1201/06/10 and DRB1*1401/54. The allele frequency distribution in the healthy controls (n = 50) was very similar to that reported in a sample of Cameroonese students [18]. The seven most frequent alleles (DRB1*0301, *0302, *0804, *1101, *1301, *1302, and *1503) accounted for 77.5% of the alleles in our control group, as compared to 71.5% in the published cohort [18]. In our study group the SE was represented by only four alleles: DRB1*0101, *0102, *0405, and *1001 (Table 3). One copy of the SE was detected in 17 RA patients (30%), 7 patients with other rheumatic diseases (14%), and 5 healthy individuals (10%) (*P *= 0.029, Chi2 test). Two copies were detected in two RA patients but in none of the controls (Table 3). HLA-DRB1*0102, *1001, *0405 were the most frequent SE-positive alleles in RA patients and in control patients.

**Table 3:** Shared epitope related HLA-DR distribution in RA patients and controls

DRB1*	RA N = 56	IRD N = 51	HI N = 50
0101	1	0	0
0102	5	4	3
1001	5	1	2
0405	4	2	0
0102/0405	1	0	0
0102/1001	1	0	0

**Total:**	**17**	**7**	**5**

HI, healthy individuals; IRD, inflammatory rheumatic diseases; RA, rheumatoid arthritis

We examined the association between the presence of the SE and that of ACPA. We observed a positive trend between the presence of SE and anti-CCP2 and anti-CCP3 (Table 4). In addition, in a univariate logistic regression, the association between the SE and the diagnosis of RA disappeared when the presence of ACPA was taken into account in the model, thus further supporting the relationship between SE and ACPA-positive RA.

**Table 4:** Role of the shared epitope as predictor of the presence of ACPA and RF in RA patients

SE (1 or 2 copies)	IgM RF		IgA RF		anti-CCP2		anti-CCP3	
	pos	neg	pos	neg	pos	neg	pos	neg
0	29	10	32	7	30	9	30	9
1	14	3	15	2	16	1	16	1
	*P *= 0.73		*P *= 0.71		*P *= 0.25		*P *= 0.12	

Patients with RA were separated according to the presence (1) or absence (0) of one or two copies of the shared epitope (SE) and the presence (pos) or absence (neg) of IgM rheumatoid factor (RF), IgA RF, anti-cyclic citrullinated peptides (CCP)2, anti-CCP3. The statistical analysis was performed by using Fisher's exact

All the immunological tests were significantly correlated to each other and the agreement ranged between 70% and 90% (kappa test). In a multivariate logistic regression analysis, IgM RF, IgA RF, anti-CCP2, and anti-CCP3 were independently associated with RA, which suggests that all these autoantibodies provide complementary information for the diagnosis of RA.

## Discussion

Our study of African RA patients confirmed previous studies in patients of European ancestry showing that anti-CCP2 and anti-CCP3 antibodies exhibit high diagnostic specificity for RA with anti-CCP2 antibodies having the highest PPV and NPV. However, the number of patients and controls included in our study limits the interpretation of these results, and future studies including a larger number of individuals should be carried out in the African population to confirm these findings. Interestingly, a recent report including Dutch patients with undifferentiated arthritis and comparing anti-CCP2, anti-CCP3, anti-citrullinated vimentin, and RF showed that anti-CCP2 tended to achieve the highest PPV for RA development [19].

IgM RF is the only serological marker for RA currently available in Cameroon. The validation of the assay for the Cameroonese population led to a marked increase of cut-off values, as compared to those recommended by the vendor, in order to improve the specificity of the test. The presence of elevated background levels of IgM RF in African controls (16% of IgM RF-positive healthy controls when using the recommended cut-off values) is probably caused by non-specific activation of the immune system by different infectious and parasitic diseases. Interestingly, the results of ACPA tests, in particular anti-CCP2 positivity, were not influenced in a similar manner as IgM RF.

Only a few studies have been conducted to determine the association between HLA-DRB1 and RA in Africa. These studies included a limited number of patients and mainly referred to HLA-DR antigens detected by serological typing or by low resolution DNA typing. One study on Zimbabweans showed a higher prevalence of HLA-DR4 in RA patients, [20]. A study performed in Senegal showed that the relative risk (RR) of developing RA was significantly associated with HLA-DR10 (RR 32), but not HLA-DR4 (RR 0.8) [21]. The frequency of SE-containing HLA-DRB1 alleles was 25.2% in African Americans with RA as compared to 13.6% in healthy subjects. Thus, the SE was significantly associated with susceptibility to RA, but the percentage of SE positivity was globally lower than that reported in RA patients of European ancestry, which ranges between 50 to 70% [22,23]. The frequency of HLA-DRB1*0401, 0404, 0405, and 1001 alleles were higher among African American RA patients than in healthy controls. Of note, a higher level of European admixture was associated with a higher likelihood of carrying the SE among African Americans. More specifically, HLA-DRB1*0401 but not the other alleles encoding the SE, was significantly associated with the presence of European ancestry [24]. In another study, HLA-DRB1*0102 and HLA-DRB1*0405 were significantly more frequent among African American RA than European RA patients with odds ratio (OR) of 8.66 and 2.75, respectively. HLA-DRB1*1001 tended also to be more frequent in African American RA than European RA patients (OR 2.11). In contrast, an opposite result was found regarding HLA-DRB1*0401 with an odds ratio of 0.15 [25]. Thus, our results as well as recent reports on African American patients indicate that, although the presence of the SE is associated with RA, its frequency is much lower than that observed in patients of European ancestry, with also a distinct SE allele profile characterized in particular by a lower HLA-DRB1*0401 frequency.

RA is a clinically heterogeneous disease and there has been some speculation recently that it may comprise at least two distinct subgroups characterized by the presence/absence of ACPA. For example, the carriage of the SE appears particularly confined to anti-CCP positive RA cases [26,27]. In addition, a significant association between SE and the presence of anti-CCP2 antibodies was demonstrated in African American RA patients [24]. In our study, there was also an association between ACPA and SE. However, this association was relatively weak, probably due to the limited number of patients included in our study. The fact that ACPA are present in a similar percentage of African patients as previously reported in European patients despite a major difference in the proportion of SE-positive patients, suggests that other non-HLA genetic factors contribute to the development of RA and of these autoantibodies in African RA patients. Of note, an African specific allele of CTLA4 has recently been shown to confer protection against RA in African Americans [28].

Tobacco use was shown to be associated with the development of the disease, in particular in anti-CCP2-positive, SE-positive RA patients [26]. In one study, the conjunction of tobacco use and HLA-DRB1*0101 or *0102 was the strongest factor for the development of these antibodies [29]. With the exception of one case, none of our patients smoked, which is in line with general living habits of African women. This finding suggests that other environmental factor may be involved in the development of RA.

To our knowledge this study is the first report on combined HLA-DRB1 SE and ACPA status in an African patient cohort without known European admixture. It has, however, limitations due to the limited number of patients and controls included. In addition, the patient population is highly selected as we had only access to outpatients followed in a university hospital, representing patients from an urban setting with moderate to severe disease. However, the Rheumatology Unit is the only specialized center available for the population of Yaoundé and its suburbs and we reduce this bias by including all the RA patients attending the clinic without any further selection.

## Conclusions

Our study showed that anti-CCP2 antibodies are sensitive and specific diagnostic markers of RA also in African patients. The discrepancy between the high prevalence of ACPA-positive patients and the relatively low number of SE-positive cases as well as the relative lack of tobacco smokers suggest that other genetic and environmental factors control the development of ACPA in African RA patients.

## Abbreviations

ACPA: anti-citrullinated peptides/proteins antibodies; AUC: area under the curve; CCP: cyclic citrullinated peptides; DMARDs: disease modifying antirheumatic drugs; IRD: inflammatory rheumatic diseases; HI: healthy individuals; PPV: positive predictive value; NPV: negative predictive value; RA: rheumatoid arthritis; RF: rheumatoid factor; ROC: receiver operating curve; RR: relative risk; SE: shared epitope.

## Competing interests

The authors declare that they have no competing interests.

## Authors' contributions

MSN recruited the patients and collected the samples. MSN and CG designed the study. SB and J-MT performed the analysis. AF performed the statistical analysis. MSN and CG drafted the manuscript and all authors revised the manuscript. All authors read and approved the final manuscript.
